# Xylanase and beta-glucanase improve performance parameters and footpad dermatitis and modulate intestinal microbiota in broilers under an *Eimeria* challenge

**DOI:** 10.1016/j.psj.2023.103055

**Published:** 2023-08-22

**Authors:** Ali Daneshmand, Alip Kumar, Sarbast K. Kheravii, Guilherme Aguiar Mateus Pasquali, Shu-Biao Wu

**Affiliations:** ⁎ School of Environmental and Rural Science, University of New England, Armidale, NSW 2351, Australia; † BASF SE, 67056 Ludwigshafen, Germany

**Keywords:** exogenous enzyme, nonstarch polysaccharide (NSP), litter moisture, *Eimeria*, broiler chicken

## Abstract

Coccidiosis is an enteric disease of poultry worldwide that compromises gut health and growth performance. The current research investigated the effects of 2 doses of a multienzyme preparation on broilers' performance, gut health, and footpad dermatitis (**FPD**) under an *Eimeria* challenge. A total of 512 mixed-sex day-old chicks (Cobb 500) were randomly allocated to 4 treatments of 8 replicates. Treatments were: 1) nonchallenged control (**NC**); 2) NC + *Eimeria* challenge (**CC**); 3) CC + recommended level of xylanase and glucanase (**XG**, 100 g/t feed [on top]); 4) CC + double XG (2XG, 200 g/t feed). *Eimeria* spp. vaccine strains were gavaged on d 9 to induce coccidiosis in chickens. Performance parameters were evaluated during starter, grower, and finisher phases, and 4 birds per pen were euthanized on d 16 for sampling, FPD was scored on d 35, and litter moisture was analyzed on d 17 and 35. The data were analyzed using 1-way ANOVA with Tukey's test to separate means, and Kruskal-Wallis test was used for non-normally distributed parameters. The results showed that the *Eimeria* challenge was successful based on reduced weight gain and feed intake during grower phase, and higher FITC-d concentration, lesion score (female), and oocyst counts (d 14) in CC group compared to N.C. group, while XG and 2XG increased (*P* < 0.001) weight gain and improved FCR compared to CC and NC groups during finisher phase. The addition of X.G. and 2XG decreased litter moisture (*P* = 0.003) and FPD (*P* < 0.001) in challenged broilers compared to the N.C. group (d 35). Supplementing XG and 2XG reestablished the population of *Lactobacillus* in the cecum of challenged birds to an intermediate level between the NC and CC groups (*P* > 0.05). The inclusion of XG tended to increase the expression of *Junctional adhesion molecule 2* (**JAM2**), which was not different from CC and NC groups (*P* > 0.05). In conclusion, the combination of xylanase and glucanase (Natugrain TS) improved the performance and modulated jejunal microbiota of broilers under mild *Eimeria* challenge.

## INTRODUCTION

A healthy gut is an essential factor for better broiler performance, especially in the postantibiotic era (Choct, 2009; Oviedo-Rondón, 2019), which is characterized by a harmonious balance of various intestinal physiological functions, including digestion, absorption, energy metabolism, microbiome stability, mucosal development, immunity, and barrier integrity (Kogut and Arsenault, 2016). Therefore, any factors compromising gut homeostasis could negatively impact chickens' immune systems and productivity (Oviedo-Rondón, 2019). One of the dominating threats to poultry gut health is coccidiosis, a globally widespread parasitic disease caused by the protozoan *Eimeria* spp. It was reported that *E. necatrix* and *E. tenella* are known as the most pathogenic species (Williams et al., 2009), while *E. acervulina, E. maxima*, and *E. tenella* are generally the most prevalent ones (Clark et al., 2016; Hauck et al., 2019). *Eimeria* spp. develop their life cycles in the host intestine and disintegrate the epithelial cells during their maturation stages resulting in chickens having reduced feed intake, poor FCR, and higher morbidity and mortality (Adedokun and Adeola, 2017; Kim et al., 2017). Furthermore, the rupture of epithelial cells leads to the leakage of plasma proteins into the intestinal lumen, promoting the proliferation of *Clostridium perfringens*, which is the main cause of necrotic enteritis in broilers (Prescott et al., 2016). Literature has shown that the negative impacts of coccidiosis on poultry in recent decades are a major issue economically for the industry. Most recently, Blake et al. (2020) reported that the economic cost of coccidiosis in chickens has exceeded U.S. $14.5 billion, including all morbidity, mortality, and veterinary management costs. Therefore, many substances such as anticoccidial drugs, prebiotics, probiotics, essential oils and enzymes have been tested and suggested to prevent, control and treat poultry coccidiosis (Bozkurt et al., 2014; Quiroz-Castañeda and Dantán-González, 2015). Among these products, exogenous enzymes have shown potential due to their possible modes of action on the host's gut physiology, microflora, and nutrient digestion.

Since the main components of poultry diets comprise cereals and soybean meal, containing various levels of factors compromising nutrients absorption like nonstarch polysaccharide (**NSP**), trypsin inhibitor, phytate, and so on, supplementing exogenous enzymes to the poultry diets to digest nutritional (starch, protein, lipid), non-nutritional (cellulose), and antinutritional (β-glucans, phytate) substrates has been a necessary practice in the poultry industry (Ravindran, 2013). Furthermore, previous studies demonstrated the beneficial effects of exogenous enzymes on reducing the activity of pathogenic *Campylobacter jejuni* and *Salmonella* Enteritidis in broilers (Bedford, 2000; Fernandez et al., 2000). However, there are significant inconsistencies between the results of studies considering the effects of different enzymes on chickens' health and performance under mild *Eimeria* challenge. For example, some researchers reported that supplementing combined enzymes (Bozkurt et al., 2014), enzymes plus probiotics (Dersjant-Li et al., 2016), phytase (Adedokun and Adeola, 2017; Shi et al., 2022), and protease (Peek et al., 2009) alleviated the negative effects of *Eimeria* challenge on growth performance and nutrient digestibility in the intestine of broiler chickens, while others reported that dietary enzyme supplementation did not control the negative effects of coccidiosis in chickens (Parker et al., 2007; Walk et al., 2011). While most previous research studied the effects of individual enzymes on *Eimeria* challenge in chickens, there is scarce information about the effects of multienzyme preparation with different doses on controlling coccidiosis in broilers so far. Therefore, the current study aimed to examine the effects of a combination of xylanase and beta-glucanase on growth performance, litter moisture, footpad dermatitis (**FPD**), intestinal integrity, digesta viscosity, gene expression, and cecal microbiota in broilers under mild *Eimeria* challenge. The study had 2 hypotheses, as follows: 1) the addition of a recommended dose of xylanase and beta-glucanase can mitigate the negative effects of coccidiosis in broilers by increasing nutrient availability of diets formulated with high NSP ingredients such as rye and barley, resulting in a recovered intestine, increased feed efficiency and consequently higher productivity, 2) a higher dose of the enzyme (double dose) can result in improved effects compared to the recommended dose.

## MATERIALS AND METHODS

The University of New England's (**UNE**) Animal Ethics Committee reviewed and approved the experimental procedures of the current study (ARA21-104).

### Exogenous Enzymes Specifications

A commercial multienzyme preparation was examined in this trial. The mixture was the combination of endo-1,4-xylanase (EC 3.2.1.8) and endo-1,4-β-glucanase (EC 3.2.1.4) (Natugrain TS, BASF SE, Ludwigshafen, Germany), providing 5,600 U xylanase and 2,500 U glucanase per gram. Activity levels of enzymes in final feed samples were measured at BASF SE (Ludwigshafen, Germany), as shown in Table 1. One xylanase unit is defined as the amount of enzyme that released 5 micromole reducing sugars, measured as xylose equivalents per minute from a buffer solution containing 1 g arabinoxylan per 100 mL (pH 3.5) at 40°C. One glucanase unit is defined as the amount of enzyme that released 1 micromole of reducing sugars, measured as glucose equivalents per minute from a buffer solution containing 0.714 g β-glucan per 100 mL (pH 3.5) at 40°C.

**Table 1:** Description of treatments, enzyme amounts, activities, and recoveries.

Treatment^1^	*Eimeria* challenge	Natugrain TS						
		Amount (g/ton feed)	Xylanase activity (U/kg feed)^4^			Glucanase activity (U/kg feed)		
			Starter (d 0–8)	Grower (d 8–19)	Finisher (d 19–35)	Starter (d 0–8)	Grower (d 8–19)	Finisher (d 19–35)
Nonchallenged control	No	-	<LOQ^2^	<LOQ	<LOQ	<LOQ^3^	<LOQ	<LOQ
Challenged control (CC)	Yes	-	<LOQ	<LOQ	<LOQ	<LOQ	<LOQ	<LOQ
CC + XG	Yes	100	306 [54.7]	325 [58.0]	432 [77.2]	173 [69.2]	162 [64.8]	323 [129.2]
CC + 2XG	Yes	200	797 [71.2]	789 [70.5]	915 [81.7]	490 [98.0]	430 [86.0]	644 [129.0]

1 Treatment abbreviations: XG: 100 g Natugrain TS (xylanase + glucanase)/ton feed, 2XG: 200 g Natugrain TS/ton feed.

2 The limit of quantification (LoQ) for xylanase was 36 units/kg.

3 The limit of quantification (LoQ) for glucanase was 49 units/kg.

4 The values in the bracket [] are the recovery percentage.

### Birds and Housing Management

A total of 512 as-hatched 1-day-old Cobb 500 broiler chicks were sourced from a commercial hatchery (Baiada Pty Ltd., Tamworth, NSW, Australia). Chicks were weighed on arrival and randomly assigned to 4 treatments with 8 pens containing 16 birds in each pen. On d 5, 2 feathers were sampled from 8 birds per pen to extract DNA to determine the sex of birds using high-resolution melting (**HRM**) analysis as described by England et al. (2021) so that the labeled birds could be used for sampling with known sex. Pens were filled with wood shavings (approximately 7–8 cm) and equipped with tube feeders and nipple drinkers. The experimental room was environmentally controlled by automatic equipment, which was set based on the lighting, temperature, and ventilation programs of Cobb-Vantress (2018b). Birds had free access to feed and water for the whole experiment period (d 0–35).

### Experimental Design and Diets

Treatments and inclusion rate of each combination of enzymes were as follows: 1) nonchallenged control (**NC**, unchallenged birds fed wheat-SBM based diet as a basal diet); 2) challenged control (**CC**, *Eimeria* spp. challenged birds fed wheat-SBM based diet as a basal diet); 3) CC + recommended level of combined xylanase and glucanase (**XG**, 100 g/t feed, Natugrain TS, BASF); 4) CC + double recommended level of xylanase and glucanase (2XG, 200 g/t feed) (Table 1). Enzymes were added on top in the experimental phases, including starter (d 0–8), grower (d 8–19), and finisher (d 19–35).

Feed ingredients were analyzed using NIRS (Adisseo PNE, Antony, France) to determine the nutrient contents of ingredients such as crude protein, amino acids, crude fiber, and crude fat before diet formulation (Supplementary File 1). All experimental diets were based on wheat, soybean meal, barley, and rye (Table 2), supplemented with phytase at 500 FTU/kg considering the matrix values, isocaloric and isonitrogenous, and formulated to meet or exceed the minimum nutritional recommendations of Cobb 500 broilers (Cobb-Vantress, 2018a), and passed through a cold pellet press (Palmer Milling Engineers Pty. Ltd., Griffith, NSW, Australia) to provide crumble diet for starter and pellet diet for grower and finisher.

**Table 2:** Composition of experimental diets.

Ingredients (as-fed basis, %)	Starter (d 0–8)	Grower (d 8–19)	Finisher (d 19–35)
Wheat	46.8	49.1	54.3
Soybean meal (CP 46%)	30.5	25.2	19.8
Barley	10.0	10.0	10.0
Rye	7.00	10.0	10.0
Canola oil	2.20	2.40	2.81
Limestone	1.15	1.10	1.03
Dicalcium phosphate	0.817	0.682	0.496
DL-methionine	0.356	0.335	0.319
L-lysine HCl 78.4	0.318	0.347	0.363
Salt	0.280	0.265	0.255
L-threonine	0.204	0.170	0.157
Na bicarbonate	0.100	0.125	0.148
UNE trace minerals^1^	0.080	0.080	0.080
UNE vitamin conc^2^	0.075	0.075	0.080
Choline chloride (60%)	0.060	0.083	0.104
Phytase^3^	0.005	0.005	0.005
Sand^4^	0.020	0.020	0.020
Total	100.0	100.0	100.0
Calculated nutrients^5^			
AMEn, kcal/kg	2925	2975	3050
Crude protein, %	22.5	20.5	18.5
Crude fiber, %	3.10	2.99	2.88
Ether extract, %	3.54	3.76	4.18
Dig. lysine, %	1.22	1.12	1.01
Dig. methionine, %	0.622	0.578	0.540
Dig. Met + Cys, %	0.910	0.850	0.795
Dig. Arginine, %	1.26	1.12	0.968
Dig. Threonine, %	0.830	0.730	0.650
Calcium, %	0.900	0.840	0.760
Available phosphorus, %	0.450	0.420	0.380
Sodium, %	0.180	0.179	0.180
Chloride, %	0.286	0.286	0.288
Linoleic acid, %	1.32	1.38	1.50
Choline, mg/kg	1718	1700	1700

1 Mineral premix provided the following per kilogram diet: Cu sulfate, 16 mg; Mn sulfate, 60 mg; Mn oxide, 60 mg; I (iodide), 0.125 mg; Se (selenite), 0.3 mg; Fe sulfate,40 mg; Zn oxide and sulfate, 100 mg.)

2 Vitamin premix provided the following per kilogram diet: vitamin A, 12,000,000 IU; vitamin D, 5,000,000 IU; vitamin E, 75 mg; vitamin K, 3 mg; cyanocobalamin,0.016 mg; folic acid, 2 mg; riboflavin, 8 mg; pyridoxine, 5 mg; biotin, 0.25 mg; thiamine, 3 mg; nicotinic acid, 55 mg; pantothenic acid, 13 mg and antioxidant ethoxyquin,50 mg.

3 Phytase: Natuphos E 10000G, 500 FTU/kg (50 g/ton).

4 Sand was replaced with the required amount of enzymes and added on top.

5 Nutrient contents of major ingredients were measured prior to the onset of the trial using near-infrared spectroscopy (NIRS, Adisseo, Antony, France) and a copy of the results was provided in a supplementary file 1.

### Eimeria Challenge

On d 9, all birds in the challenge groups were gavaged with 1 mL of live sporulated strains containing *Eimeria acervulina* (5,000 oocysts), *Eimeria maxima* (5,000 oocysts), and *Eimeria brunetti* (2,500 oocysts) provided by Eimeria Pty Ltd (Ringwood, VIC, Australia), while nonchallenged birds received the same amount of sterile phosphate buffer solution.

### Performance Parameters

Since the challenge was induced on d 9, birds in NC and CC pens were considered as 1 group until d 8 (end of starter phase). All birds and the remaining feed of each pen were weighed at the end of starter (d 8), grower (d 19), and finisher (d 35) to calculate performance parameters, including weight gain, feed intake, and feed conversion ratio (**FCR**) for each phase, and all data were used to calculate the parameters of the whole period (d 0–35). The number and weight of dead birds were recorded daily to correct feed intake and FCR accordingly. Necropsy was carried out to examine the cause of death and all the dead, sampled, and birds left on d 35 were opened to determine the sex by visual inspection of testes.

### Sampling and Intestinal Lesion Score

On d 16, 4 birds (2 males and 2 females identified by DNA sexing) per pen were electrically stunned, blood samples were collected via jugular vein, and then carcasses were dissected to collect samples. The intestine was carefully separated from the carcass and divided into duodenum, jejunum, and ileum to score coccidiosis lesions based on a scale of 0 (none) to 4 (extensive coalescence of lesions with thickening of the wall) as described previously (Johnson and Reid, 1970). The ileal contents of male and female birds and the cecal contents of male birds were gently collected into the sterile tubes kept in liquid nitrogen and then preserved at −20°C for subsequent digesta viscosity analysis and DNA extraction for microbiota quantification, respectively. Two sections of proximal jejunum tissue (2 cm) of male birds were separated, rinsed in cold phosphate-buffered saline (**PBS**), immediately placed in 2 mL safe-lock Eppendorf tubes containing RNAlater, kept in a fridge for 4 h, and preserved at −20°C for subsequent RNA extraction.

### Intestine Permeability

Fluorescein isothiocyanate dextran (**FITC-d**, Sigma-Aldrich, Stockholm, Sweden) was used to determine gut permeability following the procedure previously described by Barekatain et al. (2019). On d 16, 2 males and 2 females per pen were gavaged with FITC-d (average molecular weight of 4,000, Sigma-Aldrich, Stockholm, Sweden) 2 h before sampling. Blood samples were collected and centrifuged at 3,000 × *g* for 15 min to obtain serum samples. The samples were diluted (1:1 v/v) with PBS for further analysis. The fluorescent levels in the serum samples were measured with an excitation wavelength of 485 nm and an emission wavelength of 528 nm on a microplate reader SpectraMax M2e (Synergy HT, Molecular Devices, San Jose, CA). The concentration of FITC-d (µg/mL) in serum samples was calculated based on a standard curve obtained with standard FITC-d concentrations following the procedures previously described by Prado-Rebolledo et al. (2017).

### Eimeria Oocyst Count

Excreta sample preparation was performed using the modified McMaster egg counting technique previously described by Kumar et al. (2022). Fresh excreta samples were collected from all pens on d 5 postchallenge (d 14) and stored at 4°C for the differential enumeration of *Eimeria* oocyst. One hundred milligrams of excreta samples were diluted with 900 μL saturated salt solution, vortexed to thoroughly mix and left for 2 h in the fridge to float oocysts and to settle sample debris. Then, 600 μL saturated salt solution was added to the Whitlock chamber (Whitlock universal slides, JA Whitlock & Co., NSW, Australia), and 150 μL of diluted samples were pipetted and added to the Whitlock chamber. The oocysts were differentially counted based on size and shape as described by Conway and McKenzie (2007) and Cervantes et al. (2020) under a microscope with a 40 × objective lens (Nikon Eclipse Ci-l, Tokyo, Japan). The counts were multiplied by 100 as the dilution factor and expressed as oocysts per gram (**OPG**) of excreta samples.

### Litter Quality, FPD, and Digesta Viscosity

On d 17 and 35, approximately 1 kg of litter samples was collected into plastic bags from 6 points (around feeder, drinkers, and end points) within each pen. The samples were pooled and weighed before and after drying in a forced air oven at 105°C for 24 h. The moisture content of samples was calculated as described by Barker et al. (2013).

All individual birds in each pen were examined and scored for FPD on d 35 based on the scoring method previously established by Allain et al. (2009). A 10-point scale was considered based on the extent and appearance of lesions: ranging from 0 indicating “no lesion” to “9” most macroscopic deep lesions.

Ileal digesta viscosity was measured in duplicates for each individual sampled bird on d 16. Ileal digesta in a 2 mL tube was centrifuged at 12,000 × *g* for 10 min at room temperature. Clean supernatant was transferred to a new 1.5 mL tube, and viscosity was measured using a Brookfield DV3T Rheometer (Brookfield Ametek, Instrumentation & Specialty Controls Division, Middleboro, MA) with a CPA-40Z spindle at 35°C. Viscosity data were expressed in centipoise (**cPs**) unit (1 cPs = 1/100 dyne s/cm^2^ = 1 mPa s).

### Bacterial DNA Extraction and Quantification

The QIAamp PowerFecal QIAcube HT kit (QIAGEN GmbH, Hilden, Germany) was used to extract DNA from the cecal content of male broilers based on the manufacturer's instructions with slight modifications. Briefly, about 300 mg of glass beads (0.1 mm) and 80 mg of cecal sample were put in a 2 mL Eppendorf tube. Then, 500 µL prewarmed PW1 buffer was added followed by cells disruption by bead beater (TissueLyser II, QIAGEN, Germany) for 4 min at a frequency of 30 Hz. The tube was centrifuged at 20,000 × *g* for 1 min and about 400 µL supernatant was transferred to a new tube. Next, 150 µL C3 buffer was added to the supernatant, mixed thoroughly and incubated on MultiThermal Shaker (Benchmark Scientific Inc., Sayreville, NJ) for 5 min at 4°C. The tube was centrifuged at 20,000 × *g* for 1 min and 400 µL supernatant plus 30 µL proteinase K were transferred to a new 2 mL tube and incubated at room temperature for 10 min. Then, 1,000 µL C4 buffer and 120 µL Ethanol (96–100%) were added to the tube and vortexed briefly for 5 s. Washing buffer AW1 (500 μL), AW2 (500 μL), and ethanol (400 μL of 96–100%) were applied at independent steps to purify DNA, through centrifugation at 20,000 × *g* for 1, 3, and 1 min, respectively, to remove the wash buffer and to dry the silica membrane completely. Finally, 100 μL of Elution Buffer was used to elute DNA into a 1.5 mL Eppendorf tube. The quantity and purity of extracted DNA samples were checked on a Nanodrop 8000 spectrophotometer (Nanodrop Technologies, Wilmington, DE), and DNA with ratios of 260/280 and 260/230 higher than 1.8 were considered of high quality and stored at −20°C. The extracted DNA was diluted 20 times with nuclease-free water, and the number of bacteria (*Bacillus, Bacteroids, Bifidobacteria, Enterobacteriaceae, Lactobacillus, Ruminococcus*) and total bacteria were quantified with the SYBR Green kit (SensiFAST SYBR No-ROX, meridian Bioscience, Sydney, Australia) using qPCR machine (Rotor-Gene Q, QIAGEN GmbH, Hilden, Germany). Table 3 shows the primers used for bacterial quantification. The quantity of the bacteria was expressed as log_10_ genomic DNA copy number per gram of digesta.

**Table 3:** Sequences of primer pairs used for qPCR analysis of listed bacteria in male cecal digesta.

Bacteria	Sequence (5′ → 3′)	Ta (°C)	Product size (bp)	References
*Bacillus* spp.	F-GCA ACG AGC GCA ACC CTTGA	63	92	Han et al. (2012)
	R-TCA TCC CCA CCT TCC TCC GGT			
*Bacteroides* spp.	F-GAG AGG AAG GTC CCC CAC	63	106	Layton et al. (2006)
	R-CGC TAC TTG GCT GGT TCA G			
*Bifidobacterium* spp.	F-GCG TCC GCT GTG GGC	63	106	Requena et al. (2002)
	R-CTT CTC CGG CAT GGT GTT G			
*Lactobacillus* spp.	F-CAC CGC TAC ACA TGG AG	63	186	Fu et al. (2006)
	R-AGC AGT AGG GAA TCT TCC A			
*Ruminococcus* spp.	F-GGC GGC YTR CTG GGC TTT	63	157	Ramirez-Farias et al. (2008)
	R-CCA GGT GGA TWA CTT ATT GTG TTA A			
*Enterobacteriaceae*	F- CAT TGA CGT TAC CCG CAG AAG AAG C	63	190	Bartosch et al. (2004)
	R- CTC TAC GAG ACT CAA GCT TGC			
Total bacteria	F-CGG YCC AGA CTC CTA CGG G	63	204	Lee et al. (1996)
	R-TTA CCG CGG CTG CTG GCA C			

### Jejunal Gene Expression

The RNeasy QIAcube HT Mini kit (QIAGEN GmbH, Hilden, Germany) was used to extract RNA from jejunal tissues of male broilers following the manufacturer's instructions. The quantity and purity of extracted RNA were assessed with a NanoDrop ND-8000 spectrophotometer (Thermo Fisher Scientific, Waltham, MA), and the integrity was assayed with the Agilent 2100 Bioanalyzer (Agilent Technologies, Inc., Waldron, Germany). The RNA samples with a ratio of 260/230 being >2.0, 260/280 between 2.0 and 2.2, and an RIN number of >7 were considered of high quality. The extracted RNA was reverse-transcribed with a SensiFAST cDNA synthesis kit (meridian Bioscience, Sydney, Australia) following the manufacturer's instructions. The RNA was converted into cDNA using the real-time PCR machine (Rotor-Gene Q, QIAGEN GmbH, Hilden, Germany), and the resulting cDNA was diluted 10 times with nuclease-free water and stored at −20°C.

The primers of target genes are listed in Table 4. qPCR was performed in duplicates using an SYBR Green kit (SensiFAST SYBR No-ROX, meridian Bioscience, Sydney, Australia) with a real-time PCR machine (Rotor-Gene Q, QIAGEN GmbH, Hilden, Germany). Eight housekeeping genes were tested to select the more stable genes in response to the treatments applied in the current study using the geNorm module of qbase+ software (version 3.0, Biogazelle, Zwijnbeke, Belgium). These genes were: *Ribosomal protein L4* (**RPL4**), *β-actin, glyceraldehyde 3-phosphate dehydrogenase* (**GAPDH**), *hypoxanthine-guanine phosphoribosyltransferase* (**HPRT**), *hydroxymethylbilane synthase* (**HMBS**), *TATA box-binding protein* (**TBP**), *tyrosine 3-monooxygenase/tryptophan 5-monooxygenase* (**YWHAZ**), and *succinate dehydrogenase subunit A* (**SDHA**). The 3 most stable genes, that is, HMBS, GAPDH, and SDHA, were used as reference genes to normalize the expression levels of jejunal target genes. The resulting data were transferred to statistical software for further analysis.

**Table 4:** Sequences of primer pairs used for qPCR analysis of listed references and target genes in the jejunum of male broilers under *Eimeria* challenge.

Genes	Sequence (5′ → 3′)	Ta (°C)	Amplicon size (bp)	References
Reference genes				
*HMBS*^1^	F:GGCTGGGAGAATCGCATAGG	60	131	Yin et al. (2011)
	R:TCCTGCAGGGCAGATACCAT			
*GAPDH*	F:GAAGCTTACTGGAATGGCTTTCC	60	66	Kuchipudi et al. (2012)
	R:CGGCAGGTCAGGTCAACAA			
*SDHA*	F:ATACGGGAAGGAAGGGGTTG	60	74	Barzegar et al. (2021)
	R:TGCTGGGGTGGTAAATGGTG			
Target genes				
*ASCT1*	F:TTGGCCGGGAAGGAGAAG	60	63	Paris and Wong (2013)
	R:AGACCATAGTTGCCTCATTGAATG			
*ACACA*	F:AGACAAGGCTGCCCGTGAG	60	181	Barzegar et al. (2021)
	R:GAAATTCCCTCTTCTGTGCCA			
*APN*	F:AATACGCGCTCGAGAAAACC	60	70	Gilbert et al. (2007)
	R:AGCGGGTACGCCGTGTT			
*ATP5A1W*	F:GGCAATGAAACAGGTGGCAG	60	232	Barzegar et al. (2021)
	R:GGGCTCCAGCTTGTCTAAGTGA			
*B^0^AT*	F:GTGTTTGGAACCCTAAATAC^↓^GAGG	60	72	Kheravii et al. (2018)
	R:TAGCATAGACCCAGCCAGGA			
*b^o,+^AT*	F:CAGTAGTGAATTCTCTGAGTGTGAAGCT	60	88	Gilbert et al. (2007)
	R:GCAATGATTGCCACAACTACCA			
*CAT1*	F:CAAGAGGAAAACTCCAGTAATTGCA		75	Gilbert et al. (2007)
	R:AAGTCGAAGAGGAAGGCCATAA			
*CAT2*	F:TGCTCGCGTTCCCAAGA		67	Gilbert et al. (2007)
	R:GGCCCACAGTTCACCAACAG			
*CLDN1*	F:CTTCATCATTGCAGGTCTGTCAG	60	103	Zanu et al. (2020)
	R:AAATCTGGTGTTAACGGGTGTG			
*FFAR4*	F:AGTGTCACTGGTGAGGAGATT		-	Slawinska et al. (2019)
	R:ACAGCAACAGCATAGGTCAC			
*GLUT2*	F:GATCGTGGCACTGATGGTT	60	171	Kheravii et al. (2018)
	R:CCACCAGGAAGAC↓GGAGATA			
*IgA*	F:GTCACCGTCACCTGGACTACA	61	192	Lammers et al. (2010)
	R:ACCGATGGTCTCCTTCACATC			
*IgG*	F:ATCACGTCAAGGGATGCCCG	60	118	Zhao et al. (2013)
	R:GCATCAGCGTCACCGAAAGC			
*IgM*	F:GCATCAGCGTCACCGAAAGC 98	60	98	Lammers et al. (2010)
	R:TCCGCACTCCATCCTCTTGC			
*JAM2*	F:AGACAGGAACAGGCAGTGCTAG	60	135	Zanu et al. (2020)
	R:ATCCAATCCCATTTGAGGCTAC			
*LAT1*	F:GATTGCAACGGGTGATGTGA	60	70	Gilbert et al. (2007)
	R:CCCCACACCCACTTTTGTTT			
*MUC2*	F:CCCTGGAAGTAGAGGTGACTG	60	143	Fan et al. (2015)
	R:TGACAAGCCATTGAAGGACA			
*OCLN*	F:ACGGCAGCACCTACCTCAA	60	123	Du et al. (2016)
	R:GGGCGAAGAAGCAGATGAG			
*PepT1*	F:TACGCATACTGTCACCATCA	60	205	Guo et al. (2014)
	R:TCCTGAGAACGGACTGTAAT			
*PRKAγ2*	F:ACGCTGGAATTACAAACCTGC	60	73	Barzegar et al. (2021)
	R:ACTTGGTTGTGGTCTTGGTGG			
*y^+^LAT1*	F:TACTGAGGCTGACTGGAGGAA	62	227	Kheravii et al. (2018)
	R:ACGACGTACAGCACAAT↓ATCTGG			
*y^+^LAT2*	F:GCCCTGTCAGTAAATCAGACAAGA	60	82	Gilbert et al. (2007)
	R:TTCAGTTGCATTGTGTTTTGGTT			
*TJP1 (ZO-1)*	F:GGATGTTTATTTGGGCGGC	60	187	Zanu et al. (2020)
	R:GTCACCGTGTGTTGTTCCCAT			

1 Genes name: HMBS: *hydroxymethylbilane synthase*; GAPDH: *β-actin, glyceraldehyde 3-phosphate dehydrogenase*; SDHA: *succinate dehydrogenase subunit A*; ASCT1: *alanine, serine, cysteine, and threonine transporter*; ACACA: *acetyl-CoA carboxylase alpha*; APN, *aminopeptidase N*; ATP5A1: *ATP synthase subunit alpha*; B0AT: *solute carrier family 6, member14*, bo; +AT: *solute carrier family 7, member 9*; CAT1: *cationic amino acid transporter-1*; CAT2: *cationic amino acid transporter-2*; CLDN1: *claudin 1*; FFAR4: *free fatty acid receptor-*4; GLUT1: *glucose transporter-1*; GLUT2: *glucose transporter-2*; IgA: *immunoglobulin A*; IgG: *immunoglobulin G*; IgM: *immunoglobulin M*; JAM2: *junctional adhesion molecule 2*; LAT1: *L type amino acid transporter-1*; MUC2: *Mucin 2*; OCLN: *occluding*; Pept1: *peptide transporter-1*; PRKAγ2: *protein kinase AMP-activated noncatalytic subunit gamma 2*; y^+^LAT1: *y^+^ L amino acid transporter-1*; y^+^LAT2: *y^+^ L amino acid transporter-2*; TJP1 (ZO-1): *tight junction protein 1* (*Zonula occludens-1*).

### Statistical Analysis

All data were checked for normal distribution and analyzed using JMP 14.0 (SAS Institute, 2018). Tukey's test was used to compare differences among means of treatments, and data were considered to be statistically significant if the *P* value <0.05. Since intestinal lesion scores, differential oocyst counts, and viscosity did not distribute normally, the data were analyzed by the nonparametric Kruskal-Wallis test and the means were compared by each other using Wilcoxon method. The sex percentage was included in the model as a covariate initially, but it was not significant. Therefore, sex was not considered in the final analysis.

## RESULTS

### Enzyme Activity and Recovery

The enzyme activity and recovery rates are shown in Table 1. NC and CC groups did not show any recordable data due to the limit of quantification for xylanase and glucanase activities being 36 and 49 U/kg, respectively. The average recovery rates for xylanase in recommended and double dose were 63.3 and 74.4%, respectfully, and these rates were 87.7 and 104.3% for recommended and double dose of glucanase, respectively.

### Performance

**Figure 1:**
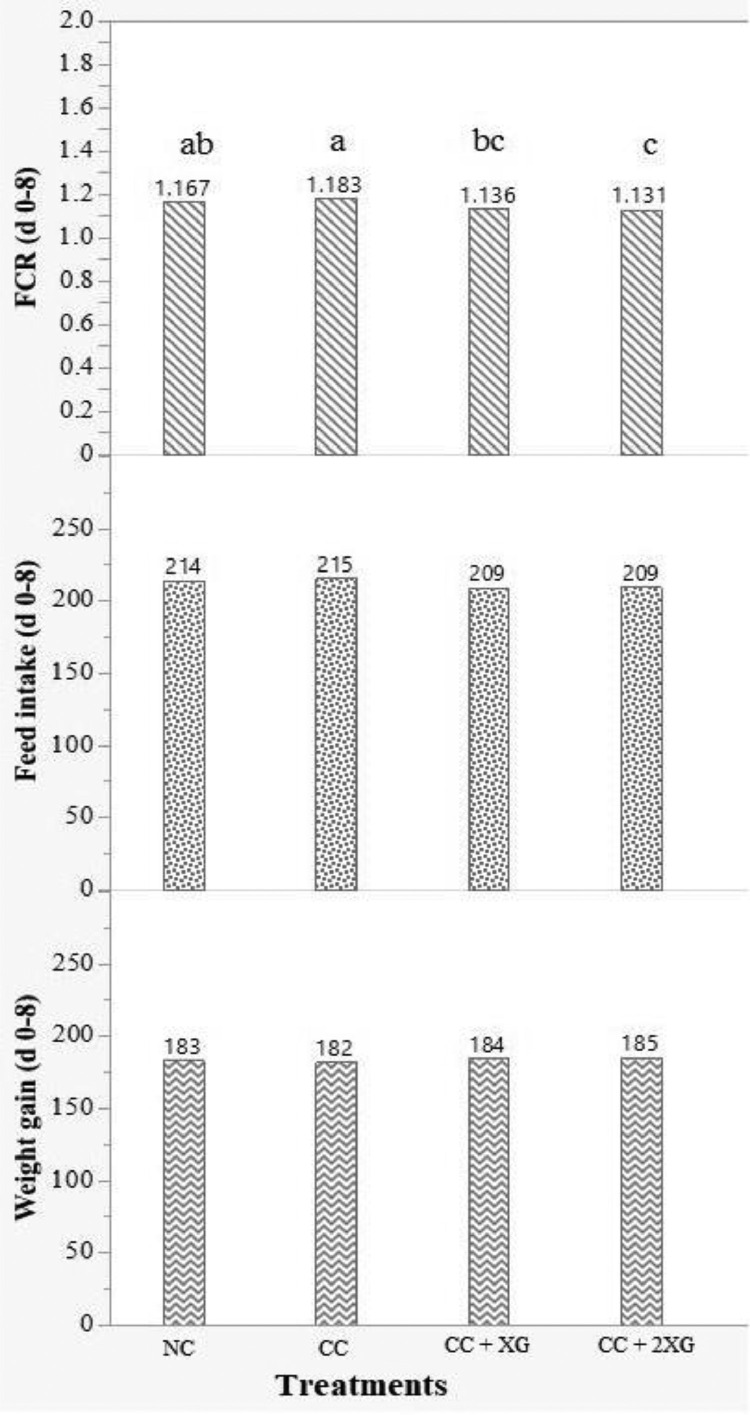
Effect of enzymes on growth performance of broilers before inducing *Eimeria* challenge (d 0–8). NC, nonchallenged birds fed wheat-SBM based diet as a basal diet; CC, challenged control (*Eimeria* challenged birds fed wheat-SBM based diet as a basal diet); XG, CC + 100 g Natugrain (xylanase + glucanase) TS/ton feed; 2XG, CC + 200 g Natugrain TS/ton feed. ^a–c^Values within a column with different letters differ significantly (*P* < 0.05).

The effects of xylanase and beta-glucanase on performance in the starter phase (d 0–8) before inducing the *Eimeria* challenge are shown in Figure 1. During this phase, XG and 2XG reduced FCR compared to control group (*P* < 0.001). Adding enzymes did not affect weight gain and feed intake during the starter phase (*P* > 0.05). At the grower phase (d 8–19), during which the challenge was induced, the *Eimeria* challenge decreased (*P* < 0.001) weight gain and feed intake and increased (*P* < 0.001) FCR in broilers compared to the NC group (Table 5). Supplementing XG and 2XG to the diet of challenged birds increased (*P* < 0.001) weight gain and reduced (*P* < 0.001) FCR compared to the CC group, although enzyme supplementation did not rehabilitate the negative effects of mild *Eimeria* challenge on weight gain, feed intake, and FCR compared to the NC group (*P* < 0.05) in the grower phase. At the finisher phase (d 19–35), while there was no significant difference in weight gain, feed intake, and FCR between the CC and NC birds, XG and 2XG increased (*P* < 0.001) weight gain and decreased (*P* < 0.001) FCR compared to CC and NC groups. The inclusion of enzymes did not affect feed intake (*P* > 0.05). Considering the whole experimental period (d 0–35), results showed that the addition of XG increased (*P* < 0.001) broilers' weight gain compared to both CC and NC groups, while 2XG had higher (*P* < 0.001) weight gain compared to CC group. Supplementing enzymes decreased (*P* < 0.001) FCR compared to CC and NC groups, albeit without showing feed intake differences during 0 to 35 d (*P* > 0.05). In addition, the results showed that inducing the *Eimeria* challenge and adding enzymes did not significantly affect mortality rate during the phases and the whole experimental period compared to the NC group (*P* > 0.05).

**Table 5:** Effect of enzymes on growth performance of broilers under *Eimeria* challenge.

Treatments^1^	Grower (d 8–19)			Finisher (d 19–35)			Whole trial (d 0–35)			
	Weight gain (g)	Feed intake (g)	FCR (g/g)	Weight gain (g)	Feed intake (g)	FCR (g/g)	Weight gain (g)	Feed intake (g)		FCR (g/g)
Nonchallenged control	689^a^	999^a^	1.452^c^	1422^b^	2864	2.020^a^	2294^b^^c^	3979		1.738^a^
Challenged control (CC)	557^c^	898^b^	1.610^a^	1487^b^	2814	1.896^a^	2227^c^	3874		1.741^a^
CC + XG	593^b^	912^b^	1.538^b^	1685^a^	2913	1.730^b^	2463^a^	3988		1.620^b^
CC + 2XG	602^b^	931^b^	1.547^b^	1662^a^	2880	1.735^b^	2448^a^^b^	3972		1.623^b^
SEM^2^	8.2	10.4	0.001	35.7	42.7	0.030	39.3	50.0		0.018
*P* value	<0.001	<0.001	<0.001	<0.001	0.437	<0.001	<0.001	0.390		<0.001

a–c Values within a column with different letters differ significantly (*P* < 0.05).

1 Basal diet contained phytase (Natuphos E 500 FTU/kg). Abbreviations: FCR, feed conversion ratio; X.G., 100 g Natugrain (xylanase + glucanase) T.S./ton feed; 2XG, 200 g Natugrain TS/ton feed.

2 SEM: standard error of means.

### Intestinal Lesion Score

Table 6 shows the effects of mild *Eimeria* challenge and enzymes on the lesion score of different segments of the broiler's intestine. Results showed that the challenge increased lesion score in the duodenum of female (*P* = 0.001) broilers compared to the NC group. The inclusion of enzymes did not mitigate the negative effects of the challenge on the duodenal lesions. The *Eimeria* challenge in the current study did not induce significant lesions in the jejunum and ileum of male and female birds (*P* > 0.05), and subsequently, dietary enzymes did not affect intestinal lesion scores in the mentioned sections compared to the NC group (*P* > 0.05).

**Table 6:** Effect of enzymes on intestinal lesion score of broilers on d 16 under *Eimeria* challenge.^1^

Treatments^2^	Duodenum		Jejunum		Ileum	
	Male	Female	Male	Female	Male	Female
Nonchallenged control	0.00^b^	0.00^b^	0.00	0.00	0.00	0.00
Challenged control (CC)	0.63^a^^b^	1.19^a^	0.06	0.44	0.06	0.06
CC + XG	0.88^a^	1.06^a^	0.19	0.25	0.13	0.00
CC + 2XG	0.75^a^^b^	1.31^a^	0.00	0.44	0.25	0.19
SEM^3^	0.295	0.316	0.103	0.188	0.137	0.140
*P* value	0.020	0.001	0.261	0.074	0.237	0.558

a–b Values within a column with different letters differ significantly (*P* < 0.05).

1 Coccidiosis lesions in different sections of the intestine were scored based on the scale of 0 (none) to 4 (extensive coalescence of lesions with thickening of the wall), as described by Johnson and Reid (1970).

2 Basal diet contained phytase (Natuphos E 500 FTU/kg). Abbreviations: X.G., 100 g Natugrain (xylanase + glucanase) T.S./ton feed; 2XG, 200 g Natugrain TS/ton feed.

3 SEM: standard error of means.

### Eimeria Oocysts Count

The results of counting different species of *Eimeria* are presented in Table 7. The challenge increased (*P* < 0.01) the number of *E. acervulina, E. brunetti*, and total oocysts in excreta samples compared to the NC group, while the challenge did not affect the count of *E. maxima* oocysts compared to the NC group (*P* > 0.05). Supplementing XG and 2XG did not affect the oocyst counts of *E. acervulina, E. brunetti*, and total oocysts (*P* > 0.05).

**Table 7:** Effect of enzymes on *Eimeria* sp. oocyst count (oocysts/g excreta) on d 14 in broilers under *Eimeria* challenge.

Treatments^1^	*E. maxima*	*E. acervulina*	*E. brunetti*	Total oocysts
Nonchallenged control	0	0^b^	0^b^	0^b^
Challenged control (CC)	13	5350^a^	2763^a^	8125^a^
CC + XG	38	8225^a^	3988^a^	12250^a^
CC + 2XG	25	7825^a^	3150^a^	11000^a^
SEM^2^	17	1611	799	2114
*P* value	0.438	0.004	0.009	0.001

a–b Values within a column with different letters differ significantly (*P* < 0.05).

1 Basal diet contained phytase (Natuphos E 500 FTU/kg). Abbreviations: X.G., 100 g Natugrain (xylanase + glucanase) T.S./ton feed; 2XG, 200 g Natugrain TS/ton feed.

2 SEM: standard error of means.

### Litter Moisture, FPD, Digesta Viscosity, and Intestinal Integrity

On d 17, mild *Eimeria* challenge increased (*P* < 0.01) litter moisture content compared to the NC group (Table 8), while XG did not lead to a difference from either CC or N.C. groups (*P* > 0.05). On d 35, while there was a significant difference between CC and NC groups (*P* > 0.05), supplementation of XG and 2XG resulted in lower (*P* = 0.003) litter moisture content compared to the NC group and had no significant difference with the CC group (*P* > 0.05).

**Table 8:** Effect of enzymes on litter moisture content, footpad dermatitis, ileal digesta viscosity, and FITC-d concentration of broilers under *Eimeria* challenge.

	Litter moisture (%)^2^		Footpad score (d 35)	Viscosity (cP, d 16)	FITC-d (μg/mL, d 16)	
Treatments^1^	D 17	D 35			Male	Female
Nonchallenged control	48.2^b^	52.6^a^	7.09^a^	21.8^a^	0.143^b^	0.185^b^
Challenged control (CC)	52.5^a^	51.1^a^^b^	7.88^a^	4.96^b^	0.279^a^	0.354^a^
CC + XG	50.8^a^^b^	48.0^b^	3.81^c^	3.55^c^	0.262^a^	0.452^a^
CC + 2XG	52.1^a^	48.3^b^	5.50^b^	2.79^d^	0.264^a^	0.352^a^
SEM^3^	0.91	0.91	0.34	0.97	0.023	0.029
*P* value	0.011	0.003	<0.001	<0.001	<0.001	<0.001

a–c Values within a column with different letters differ significantly (*P* < 0.05).

1 Basal diet contained phytase (Natuphos E 500 FTU/kg). Abbreviations: X.G., 100 g Natugrain (xylanase + glucanase) T.S./ton feed; 2XG, 200 g Natugrain TS/ton feed.

2 It should be mentioned that the wet litter of each pen was replaced with approximately 2.0 to 2.5 kg new wood shavings on d 17 after collecting samples, due to high moisture content.

3 SEM: standard error of means.

While broilers in CC and NC groups had similar FPD on d 35 (*P* > 0.05), adding XG and 2XG decreased (*P* < 0.001) the FPD score in challenged birds (Table 8). Supplementation of challenged broilers with XG showed a significantly lower (*P* < 0.001) score of FPD compared to all other groups.

The data from Table 8 present the effects of enzyme supplementation in the diet on the viscosity of ileal digesta in challenged broilers on d 16. The challenge decreased (*P* < 0.001) the viscosity of the digesta compared to the NC group. Adding XG reduced (*P* < 0.001) viscosity compared to the CC and NC groups. The supplementation of 2XG to the diet of challenged birds led to the lowest viscosity among all the groups with a significant difference (*P* < 0.001) even compared to XG group, the second lowest group.

Challenged male and female broilers in CC group showed higher (*P* < 0.001) concentrations of FITC-d in their serum samples compared to the NC group (Table 8). Adding enzymes to the diet of challenged birds did not show a difference in the concentration of FITC-d from the CC group (*P* > 0.05), although the concentration of FITC-d in enzyme groups was significantly higher than the NC group (*P* < 0.001).

### Quantification of Bacterial Groups

The results of the cecal bacterial quantification are shown in Table 9. Mild *Eimeria* challenge reduced the levels of *Bifidobacteria* (*P* = 0.006) and *Lactobacillus* (*P* = 0.024) in the cecum of challenged birds compared to the NC group. Supplementing XG to the diet of challenged broilers shifted the levels of *Bifidobacteria* and *Lactobacillus* toward the level in NC group showing no difference between them (*P* > 0.05). The inclusion of 2XG to the diet of challenged birds did not show a difference in the level of *Lactobacillus* from either CC or NC groups (*P* > 0.05). Inducing the challenge and the addition of enzymes did not affect the level of *Bacillus, Bacteroids, Enterobacteriaceae, Ruminococcus*, and total bacteria in the cecum of broilers (*P* > 0.05).

**Table 9:** Effect of enzymes on cecal microbiota (log_10_ genomic DNA copies/g digesta) of male broilers under *Eimeria* challenge.

Treatments^1^	*Bacillus*	*Bacteroids*	*Bifidobacteria*	*Enterobacteriaceae*	*Lactobacillus*	*Ruminococcus*	Total bacteria
Nonchallenged control	8.39	9.48	9.34^a^	9.09	11.67^a^	9.11	11.56
Challenged control (CC)	8.16	9.82	8.94^b^	8.93	11.34^b^	9.10	11.41
CC + XG	8.29	9.93	9.05^a^^b^	9.13	11.52^a^^b^	9.18	11.48
CC + 2XG	8.30	9.91	8.93^b^	9.12	11.60^a^^b^	9.14	11.56
SEM^2^	0.058	0.117	0.083	0.124	0.074	0.067	0.058
*P* value	0.087	0.06	0.006	0.666	0.024	0.935	0.257

a–b Values within a column with different letters differ significantly (*P* < 0.05).

1 Treatment abbreviations: XG, 100 g Natugrain (xylanase + glucanase) TS/ton feed; 2XG, 200 g Natugrain TS/ton feed.

2 SEM: standard error of means.

### Gene Expression

#### Nutrient Transporter

The effects of mild *Eimeria* challenge and enzymes on the expression of nutrient transporter genes in the jejunum of male broilers are shown in Table 10. The challenge downwardly expressed (*P* = 0.013) y^+^LAT1 in the jejunum of birds compared to the NC group, while the enzyme treatments did not mitigate the adverse effects of the challenge compared to the CC group, and had lower expression compared to the NC group (*P* = 0.013). Mild *Eimeria* challenge and adding enzymes did not change the expression patterns of all other nutrient transporter genes in the jejunum of male broilers (*P* > 0.05).

**Table 10:** Effect of enzymes on jejunal expression of nutrient absorption-related genes of male broilers under *Eimeria* challenge.

Treatments^1^	Amino acids^2^								Carbohydrates	Peptides
	ASCT1	b^o,+^AT	B^0^AT	LAT1	y^+^LAT1	y^+^LAT2	CAT1	CAT2	GLUT2	PepT1
Nonchallenged control	0.83	1.38	1.30	0.84	1.70^a^	0.99	0.96	0.94	1.36	0.96
Challenged control (CC)	1.21	1.03	1.15	1.07	0.97^b^	1.08	1.40	0.74	1.29	1.21
CC + XG	1.14	0.98	0.96	1.19	1.02^b^	1.03	1.71	1.54	1.08	1.04
CC + 2XG	1.16	1.25	1.15	1.05	0.96^b^	1.12	1.23	1.00	0.99	1.11
SEM^3^	0.209	0.146	0.169	0.134	0.172	0.111	0.293	0.385	0.176	0.204
*P* value	0.564	0.199	0.590	0.348	0.013	0.847	0.351	0.538	0.420	0.845

a–b Values within a column with different letters differ significantly (*P* < 0.05).

1 Treatment abbreviations: XG, 100 g Natugrain (xylanase + glucanase) TS/ton feed; 2XG, 200 g Natugrain TS/ton feed.

2 Genes name: ASCT1: *alanine, serine, cysteine, and threonine transporter*; B^0^AT: *solute carrier family 6, member14*; b^o,+^AT: *solute carrier family 7, member 9*; CAT1: *cationic amino acid transporter-1*; CAT2: *cationic amino acid transporter-2*; GLUT2: *glucose transporter-2*; LAT1: *L type amino acid transporter-1*; Pept1: *peptide transporter-1*; y^+^LAT1: *y^+^ L amino acid transporter-1*; y^+^LAT2: *y^+^ L amino acid transporter-2*.

3 SEM: standard error of means.

#### Digestion, Integrity, and Immunity

The current results showed that the *Eimeria* challenge reduced (*P* = 0.004) the expression of APN and JAM2 (*P* = 0.021), and increased (*P* = 0.002) the expression of FFAR4 in the jejunum of male birds compared to the NC group (Table 11). Supplementation of enzymes did not reverse the expression of APN and FFAR4 compared to the CC group, while XG did not show a difference in the expression of JAM2 from either CC or NC groups (*P* > 0.05). Mild *Eimeria* challenge and supplementing enzymes did not change the expression pattern of other genes in the jejunum of challenged birds (*P* > 0.05).

**Table 11:** Effect of enzymes on expression of jejunal digestion-, integrity- and immunity-related genes of male broilers under *Eimeria* challenge.

Treatments^1^	Digestion^2^					Integrity				Immunity			
	ACACA	APN	ATP5A1W	PRKAγ2	FFAR4	CLDN1	JAM2	OCLN	TJP1	IgA	IgG	IgM	MUC2
Nonchallenged control	0.86	2.60^a^	1.36	1.25	0.59^b^	0.96	1.70^a^	0.95	1.11	1.39	2.75	1.53	1.33
Challenged control (CC)	1.15	1.14^b^	1.00	1.31	1.28^a^	1.60	1.00^b^	1.08	1.06	0.90	1.04	0.93	1.15
CC + XG	1.18	1.07^b^	1.09	1.01	1.23^a^	1.25	1.05^a^^b^	1.12	1.13	0.52	1.25	1.04	1.01
CC + 2XG	1.04	1.18^b^	1.16	1.10	1.21^a^	1.16	1.02^b^	1.06	0.99	1.50	1.55	1.32	1.08
SEM^3^	0.117	0.254	0.115	0.340	0.128	0.394	0.173	0.125	0.145	0.334	0.488	0.289	0.123
*P* value	0.251	0.004	0.178	0.920	0.002	0.711	0.021	0.798	0.903	0.192	0.107	0.486	0.318

a–b Values within a column with different letters differ significantly (*P* < 0.05).

1 Treatment abbreviations: XG, 100 g Natugrain (xylanase + glucanase) TS/ton feed; 2XG: 200 g Natugrain TS/ton feed.

2 Genes name: ACACA: *acetyl-CoA carboxylase alpha*, APN: *aminopeptidase N*; ATP5A1: *ATP synthase subunit alpha*; CLDN1: *claudin 1*; FFAR4: *free fatty acid receptor-*4; IgA: *immunoglobulin A*; IgG: *immunoglobulin G*; IgM: *immunoglobulin M*; JAM2: *junctional adhesion molecule 2*; MUC2: *mucin 2*; OCLN: *occludin*; PRKAγ2: *protein kinase AMP-activated noncatalytic subunit gamma 2*; y^+^LAT1: *y^+^ L amino acid transporter-1*; y^+^LAT2: *y^+^ L amino acid transporter-2*; TJP1 (ZO-1): *tight junction protein 1 (Zonula occludens-1).*

3 SEM: standard error of means.

## DISCUSSION

This study examined the effects of recommended and double doses of xylanase plus glucanase (Natugrain TS) on the performance, litter quality, and markers of footpad and gut health in broilers under mild *Eimeria* challenge. It was revealed that the additions of XG and 2XG increased body weight gain and reduced FCR compared to the CC group during the 35-day experiment. Interestingly, the additions of XG and 2XG showed lower FCR than the NC group. In addition, the inclusion of XG and 2XG in the diet of challenged broilers reduced litter moisture content and FPD score. Furthermore, XG shifted the level of *Bifidobacteria* and *Lactobacillus* and the expression level of JAM2 gene toward the nonchallenged birds. Therefore, we accept the hypothesis that the supplementation of xylanase plus beta-glucanase is beneficial to intestinal health and can help to mitigate the negative effects of *Eimeria* challenge by modifying different physiological and biochemical pathways, especially when birds are fed with diets containing high NSP levels. Also, it appeared that the double dose of XG did not show a more prominent effect compared to the recommended dose. Therefore, the second hypothesis that a higher dose of the enzyme (double dose) could result in improved effects compared to the recommended dose was rejected.

The addition of combined enzymes to the diet resulted in higher feed efficiency in the challenged broilers even when they were compared with nonchallenged birds during the 0- to 35-day trial period. The findings of previous research showed that mild *Eimeria* challenge significantly decreased weight gain and feed intake and devastated FCR in broilers, especially at early ages (Luquetti et al., 2016; Wang et al., 2019), which are in agreement with the results of the current study. Different strategies have been suggested to control coccidiosis in broilers including some studies focusing on the application of exogenous enzymes. As reviewed elsewhere (Ravindran, 2013; Alagawany et al., 2018), the beneficial effects of different exogenous enzymes on the growth performance of broilers have been attributed to 1) degradation of specific bonds in ingredients; 2) degradation of antinutritional factors; 3) disruption of endosperm cell walls to release nutrients; 4) shift of digestion to more efficient digestion sites; 5) reductions in endogenous secretions from the gut; 6) reduction in the weight of the intestinal tract; 7) changes in the intestinal microflora profile; and 8) augmentation of endogenous digestive enzymes. Bozkurt et al. (2014) examined the effects of various additives, such as pro- and prebiotics, enzymes, and essential oils, against the *Eimeria* challenge in broilers. They demonstrated that a combination of enzymes (xylanase, protease, β-glucanase, and mannanase) significantly increased body weight gain and reduced FCR compared to the challenged birds at the end of the experiment (d 42). In another coccidiosis-enzyme study, Karunaratne et al. (2021) challenged broilers with live *Eimeria* vaccine and supplemented diets with β-glucanase (0, 0.01, and 0.1%) and reported 0.1% β-glucanase significantly reduced FCR compared to the challenged group at the end of the trial (d 32). The proposed mechanism of supplementing enzymes on the performance of *Eimeria*-challenged broilers can be attributed to the ability of NSPase enzymes to reduce intestinal viscosity, release trapped nutrients from limited feed supply, and consequently support the immune response. Indeed, the immune system plays a determinant role in a trade-off between mounting an immune response and other body functions (e.g., growth and reproduction) (Doeschl-Wilson et al., 2009; van der Most et al., 2011), and is the primary receiver of more nutrients in the occurrence of any challenge to support host cells against such stimulant (Iseri and Klasing, 2014; Peebles et al., 2014). For example, it was reported that the immune system (leukocytes plus protective proteins) requires 0.4% of the body's lysine (equivalent to 5.4% lysine in a pectoralis muscle) under normal conditions, while this amount will double under a robust immune response to a challenge (Iseri and Klasing, 2014). On the other hand, it was shown that supplementing exogenous enzymes to diets containing NSPs significantly increased energy efficiency in *Eimeria*-challenged broilers (Dersjant-Li et al., 2016) and improved performance by eliminating the nutrient encapsulating effect of the cell wall and ameliorating viscosity problems (Masey O'Neill et al., 2014). Furthermore, previous research showed that although exogenous enzymes do not have direct effects on oocysts shedding, they may reduce lesion scores, especially in cecum, by changing the flow and content of nutrients for the resident microbiota in lower sections of the gut (Parker et al., 2007; Kiarie et al., 2013). This change in nutrient flow can affect the production of volatile fatty acids in GIT which may result in the improvement of lesions, rehabilitating microbiota in favor of beneficial bacteria, and mitigating the negative effects of coccidiosis (Parker et al., 2007; Peek et al., 2009). Thus, it could be postulated that the supplementation of exogenous NSPases to the diet containing high NSPs releases various trapped nutrients into the intestinal lumen of *Eimeria*-challenged broilers which in turns provides a source of nutrients for boosting the immune response during challenge and rehabilitating the microstructure of the intestine and decreases the energy requirements for immune responses during recovery phase. Therefore, the endogenous sources of nutrients originating from catabolism of vital sources may be saved for other purposes such as reproduction and growth, as demonstrated by high body weight gain and lower FCR in the *Eimeria*-challenged broilers in the current study and others (Bozkurt et al., 2014; Dersjant-Li et al., 2016; Karunaratne et al., 2021). In the present study, adding XG or 2XG increased body weight gain and reduced FCR in the *Eimeria*-challenged broilers, possibly through increasing the nutrient availability for boosting immune response and then changing the nutrient flow from the immune system to the growth in broilers.

The supplementation of enzymes modulated the population of beneficial bacteria in the cecum of *Eimeria*-challenged broilers. The chicken cecum harbors diverse communities of commensal and pathogenic bacteria, which can affect the health and growth of the host by manipulating gut pH, nutrient absorption, and mucosal immunity (Apajalahti et al., 2004). It was well-documented that *Eimeria* spp. can disturb the bacterial balance in the broiler's intestine. Indeed, host cells secrete cytokines such as IL-10 to protect themselves against *Eimeria* invasion, while this parasite exploits the IL-10 mRNA production and invades the host immune system to complete its life cycle (Sand et al., 2016; Wei et al., 2019). During the life cycle, *Eimeria* disrupts the lining of the intestinal tissue, resulting in a significant change in the profile of available nutrients for the microflora through 1) the secretion of several nutrients into the intestinal lumen from ruptured epithelial cells, and 2) undigested particles due to the dysfunctionality of intestinal cells. These phenomena fluctuate the bacteria population and allow harmful bacteria to accumulate, causing an imbalance in the gut microbiota (Madlala et al., 2021). In agreement with the current study, it was shown that *Eimeria* reduces the number of beneficial bacteria like Firmicutes (e.g., *Lactobacillus*), resulting in a loss of energy and carbon sources for the host (Forte et al., 2018) and changing the microbial community in favor of pathogenic bacteria such as *C. perfringens* that causes necrotic enteritis in chickens (Prescott et al., 2016). Parker et al. (2007) reported that the addition of a commercial combined enzyme (xylanase, protease, and amylase) to the diet of *Eimeria*-challenged broilers showed very similar G + C% profiles related to the unchallenged control. Since the major components of the feed in the current trial were wheat, barley, and rye, which are rich sources of different soluble and nonsoluble NSPs, mainly arabinoxylans and β-glucans, it can be postulated that supplementing xylanase and beta-glucanase to the diets could release more oligomers in the intestine that can play a prebiotic role for beneficial bacteria (Courtin et al., 2008). The colonization of these bacteria contributes to the production of butyrate (Wu et al., 2019), which plays crucial roles in reducing chronic inflammation, relieving the severity of *Eimeria* infection (Chen et al., 2020), stimulating cell growth in the intestinal lining (Cui et al., 2017), and serving as the energy and carbon source for growth (Pourabedin et al., 2015), as shown in the current study by increased weight gain and reduced FCR. Overall, the current study demonstrated that enzyme supplementation had the potential to manipulate the bacterial community in favor of beneficial bacteria, as evidenced by the shift of ileal *Bifidobacteria* and *Lactobacillus* population in birds supplemented with X.G. toward the nonchallenged group.

Supplementing the combinations of xylanase and glucanase (i.e., XG and 2XG) to the diet improved litter quality and lowered FPD score in *Eimeria*-challenged broilers compared to the nonchallenged group. Previous studies showed that various factors could increase litter moisture in broiler flocks, including environmental components (housing, litter materials, etc.), diet ingredients (dietary electrolytes, viscous grain, etc.), pathogenic agents causing diarrhea (*Eimeria* spp., *C. perfringens*, etc.) (Dunlop et al., 2016; Swiatkiewicz et al., 2017). It was shown that the presence of high viscous cereals in the diet of poultry increases the digesta viscosity in the intestine of birds leading to reduced digesta passage rate, sticky droppings, and consequently wet litter (Annison and Choct, 1991; Masey O'Neill et al., 2014), as observed in the current study. Indeed, nutritional and pathogenic factors were simultaneously applied in the present study, which resulted in higher litter moisture in the challenged group on d 17 and, interestingly, in the nonchallenged group on d 35. A primary consequence of wet litter in poultry flocks is FPD, characterized by lesions on the plantar surface of the feet (Greene et al., 1985). This disease can negatively affect flock profits by reducing the desire of birds to move toward feed, consequently causing a decline in animal welfare and growth performance (Shepherd and Fairchild, 2010; De Jong et al., 2014). Furthermore, since paws are high-demand edible parts of chicken at least in some countries, feet with severe dermatitis are unacceptable for human consumption resulting in significant economic losses (Shepherd and Fairchild, 2010). Previous studies demonstrated that supplementing broiler diets with enzymes (Kölln et al., 2017; Park and Sun, 2022) lowered the incidence of the disease in broilers. The exact mechanism of how enzymes affect litter moisture is not fully understood, but a mode of action has been hypothesized based on the reduced water intake by NSP-degrading enzymes in broilers. Since viscous grain-based diets increase intestinal viscosity, they reduce electrolyte absorption from the lumen, decrease water absorption, and increase water consumption, leading to wet litter (Van der Klis et al., 1993). In contrast, the addition of NSP-degrading enzymes decreases the viscosity of digesta and water intake, resulting in lower litter moisture and reduced bird FPD (Garcia et al., 2008; Shirzadi et al., 2009). While most previous studies examined the effects of enzymes on litter quality and FPD under normal conditions, the current study considered the enzyme effects on these parameters in broilers under simultaneous challenges of high NSP diet and mild coccidiosis. As observed in this study, the presence of *Eimeria* in the intestinal lumen exacerbates the negative effects of viscous grains resulting in higher litter moisture. On the other hand, the combinations of xylanase and glucanase (i.e., XG and 2XG) lowered the litter moisture in the *Eimeria*-challenged broilers, possibly by decreasing digesta viscosity and water consumption by releasing electrolytes such as Na, Mg, etc. from NSPs modifying the water flow through the intestinal lining. This also led to lowered incidence of FPD. Furthermore, XG may have regulated the production of tight junction proteins as evidenced by the shift of JAM2 gene expression toward that of the nonchallenged group. It could be postulated that XG beneficially affected gut integrity in challenged broilers, resulting in less water leakage to the intestinal lumen, lowered excreta and litter moisture, and consequently reduced FPD. Similarly, Kölln et al. (2017) concluded that adding half the recommended dose of a combined enzyme (xylanase and β-glucanase) to the diet of broilers significantly reduced digesta viscosity in the proximal section of the small intestine, lowered FCR, and significantly decreased FPD compared to the control group. Overall, the current study showed the beneficial effects of using xylanase and glucanase (X.G. and 2XG) with significant improvement in litter quality and broilers FPD.

The present study indicated that combinations of xylanase and glucanase (Natugrain TS) increased weight gain, improved FCR, and shifted *Bifidobacteria* and *Lactobacillus* levels toward the nonchallenged group, possibly through providing required nutrients for the immune system and supporting the growth of beneficial bacteria in the intestine of challenged birds. Furthermore, combinations of xylanase and glucanase improved litter quality and reduced FPD, possibly by reducing excreta moisture by changing the structure of antinutritional factors such as NSPs in the intestine of challenged broilers. Since the recommended (i.e., XG) and double recommended (i.e., 2XG) doses of enzymes showed similar effects, the single recommended dose of combined enzymes should suffice for the birds under the conditions in the current study. While the findings led to the partial acceptance of hypotheses, further research is required to find out how the combinations of enzymes regulate immune response and microbiota population for improved performance in broilers, especially under challenge conditions.

## Appendix Supplementary materials

Image, application 1

## Footnotes

Supplementary material associated with this article can be found in the online version at doi:10.1016/j.psj.2023.103055.
