# Supplementary material for: Xylanase and beta-glucanase improve performance parameters and footpad dermatitis and modulate intestinal microbiota in broilers under an Eimeria challenge
Source: Poult Sci. 2023 Aug 22;102(11):103055. doi: 10.1016/j.psj.2023.103055 (PMC10514458; doi:10.1016/j.psj.2023.103055)
Supplement: Supplementary file 1 [file mmc1.docx]

| **Supplementary file 1**. Results of NIR (Adisseo PNE, Antony, France) analysis of main ingredients before the onset of the study. | | | | |
| --- | --- | --- | --- | --- |
| **Analysis** | **Wheat** | **SBM (CP 46%)** | **Barley** | **Rye** |
| Dry Matter, g/100g | 89.9 | 91.13 | 87.38 | 88.84 |
| AMEn (kcal/kg) | 3153 | 2319 | 2737 | 3111 |
| Crude Protein, g/100g | 11.45 | 47.9 | 8.35 | 9.66 |
| Ash, g/100g | 1.58 | 6.46 | 2.07 | 1.44 |
| Crude Fiber, g/100g | 2.34 | 4.25 | 5.42 | 2.57 |
| Dig. Lysine, g/100g | 0.28 | 2.64 | 0.26 | 0.27 |
| Dig. Methionine, g/100g | 0.16 | 0.56 | 0.14 | 0.14 |
| Dig. Cystine, g/100g | 0.21 | 0.52 | 0.19 | 0.18 |
| Dig. Threonine, g/100g | 0.27 | 1.62 | 0.25 | 0.23 |
| Dig. Tryptophan, g/100g | 0.14 | 0.6 | 0.12 | 0.12 |
| Dig. Valine, g/100g | 0.43 | 1.97 | 0.37 | 0.37 |
| Dig. Isoleucine, g/100g | 0.35 | 2 | 0.3 | 0.29 |
| Dig. Leucine, g/100g | 0.69 | 3.22 | 0.59 | 0.54 |
| Dig. Phenylalanine, g/100g | 0.47 | 2.12 | 0.4 | 0.4 |
| Dig. Histidine, g/100g | 0.24 | 1.06 | 0.21 | 0.19 |
| Dig. Arginine, g/100g | 0.52 | 3.12 | 0.48 | 0.43 |
| Total Phosphorus, g/100g | 0.31 | 0.72 | 0.31 | 0.33 |
| Av. Phosphorus, g/100g | 0.18 | 0.26 | 0.18 | 0.14 |
